# A Novel Case of CMV Resistance to Valganciclovir and Maribavir in a Renal Transplant Patient

**DOI:** 10.3389/ti.2024.11985

**Published:** 2024-01-19

**Authors:** Helen Pearce, Emma K. Montgomery, Neil Sheerin, Helena Ellam

**Affiliations:** ^1^ Newcastle Hospitals Trust, Newcastle upon Tyne, United Kingdom; ^2^ Renal Services, Newcastle upon Tyne Hospitals NHS Foundation Trust, Newcastle upon Tyne, United Kingdom; ^3^ Immunity and Inflammation Theme, Faculty of Medical Sciences, Translational and Clinical Research Institute, Newcastle University, Newcastle upon Tyne, United Kingdom; ^4^ Department of Virology, Newcastle upon Tyne Hospitals NHS Foundation Trust, Newcastle upon Tyne, United Kingdom

**Keywords:** CMV infection, resistance, renal, transplant, maribavir

Dear Editors,

Cytomegalovirus (CMV) is one of the most common viruses causing infectious complications after kidney transplantation [1, 2]. Anti-viral prophylaxis and pre-emptive therapy are the mainstays of CMV prevention. Maribavir is an oral benzimidazole riboside drug thought to have potent, selective multimodal anti-CMV activity, thus conferring protection against CMV strains resistant to traditional anti-viral drugs [3]. Whilst valganciclovir remains the first line oral anti-viral treatment in the management of CMV, maribavir offers a promising oral alternative to previous second line nephrotoxic drugs, foscarnet and cidofovir [4]. Approved by the U.S. Food and Drug Administration in November 2021, maribavir has been recommended by National Institute for Health and Care Excellence (NICE) for patients with resistance to at least one other first-line medication used in the management of CMV [4, 5]. Despite maribavir’s novel mode of action, we report a case of resistance to both ganciclovir and maribavir in a patient following kidney transplantation.

A 70-year-old male with end-stage kidney disease, underwent kidney transplantation on 27/11/22. The patient was high risk for CMV disease (donor CMV seropositive/recipient CMV seronegative). Histocompatibility report issued day 44 post-transplant and showed donor/recipient serologic equivalents of HLA-B, -C, -DRB1, -DQB1, and–DPB1 antigens were matched, aside from HLA-A (donor HLA type A*02/A*24, recipient HLA type A*68). Post transplantation, the patient was initiated on CMV prophylaxis in the form of oral valganciclovir, 450 mg three times weekly for 100 days, alongside his maintenance immunosuppressant regime (tacrolimus (Adoport) 5 mg BD, mycophenolate mofetil (MMF) 500 mg BD and prednisolone 5 mg OD).

In the weeks following transplantation, the patient reported no symptoms suggestive of CMV disease. However, day 53 post-transplant, a CMV viraemia was detected from a whole blood CMV assay (viral load 72,800 IU/mL, eGFR 28, lymphocyte count 0.73 10^9^/L, tacrolimus level 13.9 ug/L) whilst the patient remained on prophylactic valganciclovir. Subsequently, the patient’s valganciclovir was increased to treatment dose (450 mg OD), MMF was suspended, tacrolimus dose decreased to 3 mg AM, 4 mg PM and prednisolone dose increased to 10 mg OD. Weekly CMV monitoring was instituted thereafter. Blood tests day 100 post-transplant showed the patients eGFR was stable at 34, the patient’s lymphocyte count was 1.14 10^9^/L and tacrolimus level 8.8 ug/L. Since CMV viral titres remained high despite being on treatment-dose valganciclovir, a sample was sent for genotypic resistance testing on day 103 post-transplant (UL54 and UL97 regions sequenced by Sanger sequencing). Results received on day 109 post-transplant identified the presence of UL97 C603W mutation (a common mutation which confers resistance to ganciclovir) and valganciclovir was subsequently stopped. The patient remained asymptomatic, however, on day 116 post-transplant blood tests showed the patient’s alanine transaminase levels were elevated at 160 IU/L, eGFR 28, lymphocyte count 0.99 10^9^/L and tacrolimus level 12.8 ug/L. In the context of increasing viral titres, maribavir therapy (400 mg BD) was initiated. It should be noted that maribavir strongly antagonises the action of valganciclovir, an effect thought to be precipitated by interference in the phosphorylation process, therefore these two medications must not be used in conjunction with one another [6]. Further amendments were made to the patient’s immunosuppressant regime, specifically a reduction in tacrolimus dose to 3 mg BD, day 116 post-transplant. In the initial phase following initiation of maribavir, the patient’s CMV viral load steadily decreased. The patient’s tacrolimus dose was further reduced to 2 mg BD in response to increasing trough levels, due to the known interaction between tacrolimus and maribavir. Blood tests day 144 post-transplant showed the patient’s eGFR remained at 28, alanine transaminase levels decreased to 88 IU/L, lymphocyte count increased to 2.23 10^9^/L and tacrolimus level 10 ug/L.

Day 165 post-transplant there was a significant rise in the patient’s CMV viral load, despite full treatment compliance (Figure 1), and a sample was sent for repeat genotypic resistance testing (UL54, UL97, UL56 and UL89 regions sequenced by Sanger sequencing). This identified the development of two new mutations in addition to the previously identified C603W mutation in the UL97 region: T409M mutation in the UL97 region (which is known to confer resistance to maribavir) and T503I mutation in the UL54 region (conferring resistance to ganciclovir and cidofovir). No drug resistance-associated mutations were identified in the UL56/UL89 regions. These results were confirmed by genotypic resistance testing at a second laboratory. Maribavir therapy was subsequently stopped on day 179 post-transplant. It should be noted that T409M is a common mutation, well described in the literature, and confers high level resistance to maribavir. Interestingly it often develops after an initial suppression of a patient’s CMV viral load, as seen in this case [7]. In the week following termination of maribavir, the patient’s CMV viral load decreased, which could be indicative of self-cleared infection. Further anti-viral treatment was not given and day 207 post-transplant, the following changes were made to the patient’s immunosuppression regime: tacrolimus (Adoport) 2 mg BD, mycophenolate mofetil 500 mg remained suspended, prednisolone reduced to 10 mg/5 mg alternate days. The patient’s kidney transplant function remains satisfactory and liver function remains stable following termination of maribavir (Day 207 post-transplant: eGFR 38, alanine transaminase 48 IU/L and lymphocyte count 2.34 10^9^/L and tacrolimus level 5.6 ug/L).

**FIGURE 1:**
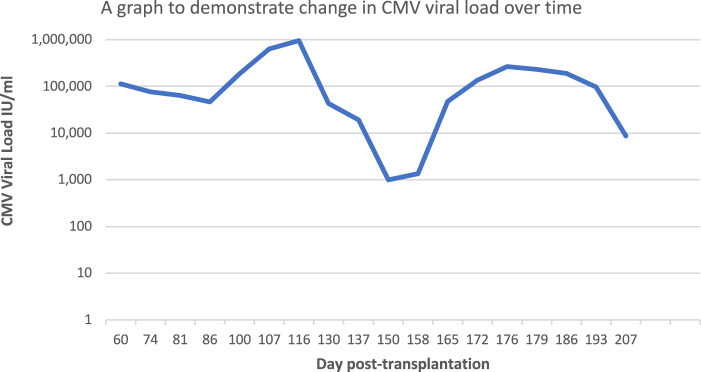
A graph to demonstrate change in CMV viral load, in-keeping with CMV resistance emergence.

In summary, to our knowledge, this is the first reported case of resistance to both valganciclovir and maribavir outside of clinical trials in the UK. Although resistance to maribavir is described in the SOLSTICE clinical trial [3, 8], our case highlights the need for clinicians to be vigilant when initiating treatment with maribavir. It should also be acknowledged that foscarnet may be a preferred option over maribavir when treating refractory CMV diseases with high viral loads [9]. A low threshold for CMV resistance testing is recommended if a patient’s CMV viral load increases whilst on treatment. Maribavir is known to increase exposure to tacrolimus [10] and our case highlights the importance of monitoring concomitant immunosuppressant blood concentration at initiation, co-administration, and discontinuation of maribavir.

## Data Availability

The original contributions presented in the study are included in the article/supplementary material, further inquiries can be directed to the corresponding author.
